# Thank you (obrigado) Cris and happy Bday (Parabéns)!

**DOI:** 10.1007/s12551-020-00712-6

**Published:** 2020-07-04

**Authors:** Vasco Sequeira

**Affiliations:** Comprehensive Heart Failure Center (CHFC), University Clinic Würzburg, Würzburg, Germany

February 2012, I attended the Biophysical Society 56th Annual Meeting in San Diego, CA, USA. Back then, I was a starting PhD student in the group of Professors Jolanda van der Velden and Ger Stienen, at the Department of Physiology of the VU Medical Center in Amsterdam (the Netherlands). I remember very vividly my first encounter with Professor Cris dos Remedios at the 56th Biophysical Meeting, because unknowingly to him (I guess up to this day) he “saved” me from a rather awkward situation while I was discussing my data with another researcher at the poster session. Let me explain, here I was presenting my work at an international meeting for the first time, in the form of a poster presentation with the unsexy title “Perturbed length-dependent activation in human HCM [hypertrophic cardiomyopathy] with sarcomere mutations in thin filament proteins.” Most of the data presented, I gathered it from isometric force experiments performed in demembranated cardiomyocytes from human HCM heart biopsies. HCM is the most common hereditary form of cardiomyopathy and is frequently caused by mutations to genes encoding cardiac sarcomeric proteins. HCM is typically characterized by hypertrophy of the left ventricle, frequently involving the septum. Septal myectomies are a successful treatment to help HCM patients, whereby the surgeon removes a portion of the septum wall that is obstructing blood flow from the left ventricle to the aortic root. In the process, I was fortunate enough to obtain unique human material to the experiments. To my “best luck,” I also worked with a very atypical human HCM cardiac biopsy that was obtained from a full heart transplant surgery, performed in a young male patient. The patient carried the homozygous cTnT-K280N HCM missense mutation. So here I was, nervous but also full of excitement at the poster session, discussing my data to another—much older—researcher. The conversation initiated smoothly and remained engaging for some minutes. But it derailed when I was asked detailed information regarding one HCM sample, in particular the homozygous cTnT-K280N biopsy. “How can you have zero information about the tissue you are working with?”—the researcher’s voice grew little by little for which he then proceeded “that is essential information for a researcher to have!”, he furiously remarked after. I could see he got upset with my answer. At that moment—I am honest—if there was a hole I could put myself in, I would have! But luckily for me, a calm and warmth voice coming towards our direction responded “that is an homozygous missense mutation in the TNNT2 gene. It is indeed a unique sample and one of its kind in the HCM world. The patient was a very young male in his 20s that required a full heart transplant!”. The other researcher was a bit confused and asked with suspicion to the approaching gentleman “how do you know about that?”—like coming straight from one of J. R. R. Tolkien’s novels (my thoughts reminded me of Gandalf the White from *Lord of the Rings*, but without the long beard) the gentleman answered with a big and open smile while turning his right hand to greet me “Well I was the one who gave the sample to Vasco! Oh and by the way, nice to meet you Vasco, I am Cris!”.

That was the first time I met Cris. That day I learnt some valuable and important lessons for my future. First, friends are great to have and we should nourish them as much as we can. They can provide you the safety, the aid, and the comfort you need. Second, with much wiser and experienced friends, there is a lot you can learn from—both at the personal and professional level. Third, I am happy that I can call Cris a friend, but also an academic Father. Fourth, I learnt from this episode that I should definitely be more cautions to my academic future regarding the material I work with during my experiments.

Thank you Cris and wishes of a great 80th birthday!

